# Outcome measures after anterior cervical decompression and fusion surgery –non-respondents do not bias the results: A Finnish spine register (FinSpine) study

**DOI:** 10.1016/j.bas.2024.104179

**Published:** 2024-12-31

**Authors:** N. Klimko, N. Danner, H. Salo, A. Malmivaara, V. Leinonen, J. Huttunen

**Affiliations:** a Department of Neurosurgery, Kuopio University Hospital and Institute of Clinical Medicine, University of Eastern Finland, Kuopio, Finland; b Finnish Institute for Health and Welfare, Helsinki, Finland; c Orton Orthopaedic Hospital, Helsinki, Finland

**Keywords:** ACDF, Anterior cervical decompression and fusion, Cervical degenerative disk disease, Non-respondent bias, Spine register, FinSpine

## Abstract

**Introduction:**

Comprehensive national spine registers are used in the Nordic countries. Register data is inherently incomplete, raising concerns about the derived results due to non-respondent bias. Few studies have addressed the effect of non-respondents on the integrity of patient-reported outcome data in national spine registers, suggesting that outcome measures after spine surgery may not differ between respondents and non-respondents.

**Research question:**

Using the Finnish national spine register (FinSpine), we aimed to assess whether non-respondents would bias patient-reported outcomes at 12 months following anterior cervical decompression and fusion (ACDF) surgery.

**Material and methods:**

FinSpine data from 5563 ACDF surgeries since 2016 were analyzed, supplemented with prescription records from the Finnish Social Insurance Institution and subcohort data from Kuopio University Hospital. Patients were grouped based on whether they completed post-operative outcome surveys. Outcomes were compared on neck and upper extremity pain, functional capacity, quality of life, sleep quality, return to work, regular use of pain medication, and opioid purchases 12 months after surgery.

**Results:**

Out of 5563 ACDF patients, 1362 (24.5%) purchased opioids during the first post-operative year. There were no significant differences in the mean cumulative opioid purchases between respondents and non-respondents. In the subcohort (n = 60), all non-respondents (n = 29) were reached and interviewed. There were no differences between respondents and non-respondents in any outcome measures at 12 months.

**Discussion and conclusion:**

Non-respondents do not bias the assessment of outcome measures following ACDF at 12 months, supporting the validity and reliability of national quality registers like FinSpine for clinical research.

## Highlights

• FinSpine is a Finnish national spine register containing data on 46800 spine surgeries, including 5563 ACDF surgeries.
• Non-respondents to patient-reported outcome surveys were studied using a subcohort, in which all patients, including non-respondents were reached for a telephone interview.
• Patient-reported outcome measures and opioid purchases did not differ between respondents and non-respondents.
• In FinSpine, non-respondents do not bias outcome measures at 12 months after ACDF surgery.

## 1 Introduction

It is a common problem for register-based observational studies to struggle with incomplete data. This issue poses significant challenges by decreasing the number of participants in the study population, hence lowering its statistical power, and can raise doubts about the validity of the data and outcomes derived from it (Thygesen and Ersbøll, 2014). For studies relying on patient-reported outcome and experience measures (PROMs/PREMs), most of the data loss is attributed to non-respondents, which can lead to bias (Patel et al., 2022). This non-respondent bias may skew interpretations and ultimately produce unreliable conclusions (Parai et al., 2020). How low a response rate is acceptable depends on the randomness of possible differences in baseline characteristics between respondents and non-respondents (Kristman et al., 2003).

A study based on the Norwegian nationwide spine register addressed non-respondent bias after cervical spine surgery, suggesting that while differences in baseline characteristics between non-respondents and respondents may exist, differences in patient reported outcome measures were not significant (Ingebrigtsen et al., 2023). Other studies based on Nordic spine registries have reported similar results in lumbar spine surgery (Elkan et al., 2018; Højmark et al., 2016).

The Finnish spine register (FinSpine) provides nation-wide data on spine surgeries performed in Finland. In our study, we aimed to investigate non-respondent bias on outcome measures after anterior cervical decompression and fusion (ACDF) surgery using FinSpine augmented with nation-wide data from the Finnish Social Insurance Institution (SII) for objective outcome data and single center subcohort data from Kuopio University Hospital.

## 2 Materials and methods

### 2.1 Data sources

#### 2.1.1 FinSpine

FinSpine is a Finnish national quality register for spine surgery containing data on spine surgeries performed in Finland since 2016. In the Finnish public healthcare system, which is tax-funded and accessible for all citizens, the vast majority of spine surgery is performed in public hospitals. The current version of the register includes all 23 Finnish public hospitals and major private hospitals (Marjamaa et al., 2023).

FinSpine is curated by the Finnish Institute for Health and Welfare (Finnish abbreviation, THL), an independent expert agency working under Finland's Ministry of Social Affairs and Health, which is responsible for pooling and storing the data from all participating centers. THL is collaborating with researchers and clinical experts to further develop the register while facilitating clinical research.

FinSpine is designed to automatically collect basic information about surgical procedures, such as date and time, and potential implants from the electronic patient records of participating hospitals. It also includes specific details about the surgical procedure: Is the surgery primary, secondary or a revision, information about pre-operative paresis and whether it is caused by medullopathy or nerve root compression, exact surgery type and diagnosis, antibiotic and thrombosis prophylaxis, levels of fusion and decompression, fusion materials used and potential in-hospital and late-occurring complications, which are entered by a surgeon. Additionally, FinSpine gathers patient-reported data on relevant medical history and responses to PROM/PREM questionnaires, including Visual-Analog scale (0–100), Neck-Disability Index (NDI) (Widbom-Kolhanen et al., 2022), Oswestry disability index (ODI) (Fairbank and Pynsent, 2000) and EQ-5D (EuroQol, 1990).

#### 2.1.2 Finnish Social Insurance Institution (SII) data

SII maintains a register of all prescription drugs purchased in Finland since 1994. We extracted all purchases of drugs containing opioids made by patients from the FinSpine ACDF population. To match each patient with one surgery date in order to calculate correct time difference between drug purchase and the date of the surgery, we had to separate patients who had no previous ACDF surgery (Primary ACDF), and those who had (Non-primary ACDF). We included drug purchases containing Morphine, Hydromorphone, Oxycodone, Buprenorphine, Codeine and Tramadol as those are the opioid drugs commonly prescribed in Finland. Opioid dosages were converted to Morphine milligram equivalents (MME) using conversion factors provided by CDC in 2022 (Dowell et al., 2022). As for Buprenorphine, conversion factors were removed in 2017. We used prior factors of 12.6 and 30 for transdermal (μg/hr) and oral Buprenorphine MME, respectively. For each purchase, total amount of opioids purchased was calculated using the following formula:

Opioidspurchased(MME)=Dispensedamount∗Drugdosage∗Conversionfactor

#### 2.1.3 Subcohort data from Kuopio University hospital

Kuopio University Hospital is one of the five university hospitals in Finland and has been taking part in FinSpine since 2017.

As a routine clinical practice at the time, at 3 and 12 months after ACDF surgery, patients were scheduled for a telephone follow-up with a neurosurgical nurse, during which the completion of self-assessment surveys (PROM/PREM's) linked to FinSpine were inquired about.

During the telephone follow-up, all patients were given a structured questionnaire for outcome measures (see Supplementary 1). Outcome measures such as radicular pain, neck pain, quality of life, functional capacity and quality of sleep were measured on a 7-point Likert scale. Daily need for painkillers and return to work -status were inquired dichotomously (yes/no).

### 2.2 Study setting and patients

**Fig. 1:**
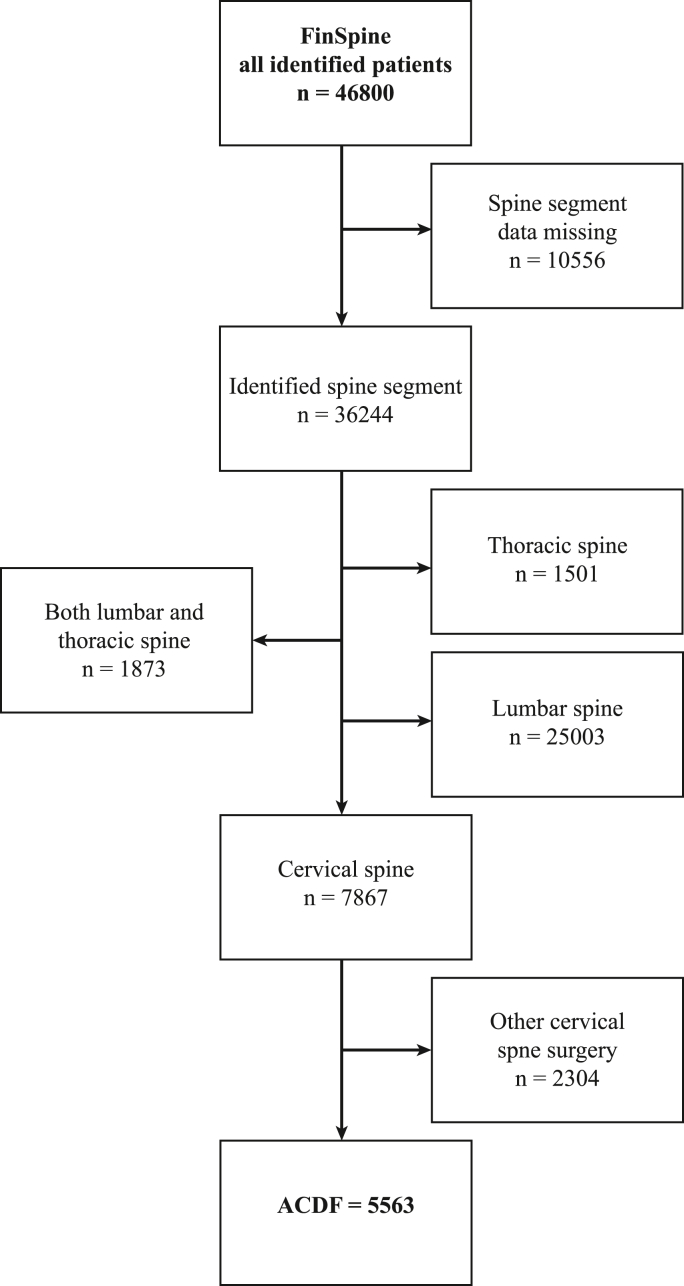
Flowchart of the FinSpine register population.

At the time of data extraction, FinSpine contained records of patients who underwent any spine (cervical, thoracic, lumbar) surgery between June 9th, 2016, and March 31st, 2023. FinSpine had entries from 19 out of 23 public hospitals, comprehensively including every center in Finland in which ACDF surgery is performed. Hospitals that perform ACDF surgery had begun gathering PROM/PREM surveys between 0 and 29 months (with an average of 10 months) following the initiation of FinSpine use. For this study, all patients who underwent ACDF surgery were selected. Out of the 46,800 identified patients in the register, 11.9% (n = 5563) received ACDF surgery (Fig. 1, Flowchart).

All consecutive patients who underwent ACDF surgery at Kuopio University Hospital in question between August 1st, 2019, and December 31st, 2019, were identified (n = 60) and all 60 patients were reached for a clinical follow-up via telephone approximately one year after the surgery.

ACDF patients from FinSpine and the single center subcohort were classified into groups depending on their status of completing the 12-month post-operative self-assessment surveys used in FinSpine. Completion of the surveys in the register population was assessed directly from register data, and for the subcohort, recorded responses from the 12-month follow-up questionnaire were used. This led to classifying the register population into 3 groups: “Not Completed”, “Partly Completed” “Fully Completed”, and the subcohort into 2 groups: “Not Completed” and “Completed”.

For all patients included, available descriptive data were gathered from the FinSpine register.

### 2.3 Data preparation and statistical analysis

Datasets were unified and prepared for analysis using Python (version 3.12.2) and the pandas data analysis library.

For statistical analysis IBM's SPSS software (version 27) and RStudio (version 2024.04.2) was used. The FinSpine data, confirmed to be normally distributed via the Kolmogorov-Smirnov test, included continuous variables presented as means with 95% confidence intervals and categorical variables displayed as frequencies and percentages. To compare group differences, one-way ANOVA was applied for continuous data, while Fisher's exact test was used for categorical data. For the SII's aggregated drug purchase data, confirming to a lognormal distribution, data is presented as medians with inter-quartile ranges and Kruskal-Wallis test was used. For all data, statistical significance was established at p < 0.05.

### 2.4 Ethical considerations

This register study has been permitted by the Finnish Institute for Health and Welfare with the approval from the Ministry of Social Affairs and Health of Finland. Record number: THL/216/6.02.00/2024. Register based studies do not require separate institutional ethical board approval according to the Finnish research legislation.

## 3 Results

The response rate to FinSpine 12-month self-assessment surveys for ACDF patients was 39.6% (n = 2201). Out of them, 67.0% (n = 1474) had completed all and partial completion was observed in 33.0% (n = 727). All consecutive (n = 60) subcohort patients were reached. According to the nurse interview, their response rate to FinSpine-related self-assessment surveys at the 12-month follow-up was 51.7% (n = 31).

### 3.1 Baseline characteristics

Baseline characteristics of both study populations are presented in Table 1 (FinSpine population) and Table 2 (Subcohort). In the subcohort, females and patients who reported regular use of painkillers pre-operatively were statistically more likely to complete the surveys, 64.5% (n = 20) vs 37.9% (n = 11) p = 0.039, 66.7% (n = 12) vs 26.7% (n = 4) p = 0.022, respectively.

**Table 1:** FinSpine ACDF population baseline characteristics.

Baseline characteristic		FinSpine 12-month PROM/PREM's self-assessment surveys					
		Not completed	Partly completed	Fully completed	Total	Missing %	p-value
**Patients, count (%)**		3362 (60.4%)	727 (13.1%)	1474 (26.5%)	5563 (100%)		
**Gender, count (%)**	**Female**	1521 (45.2%)	357 (49.1%)	686 (46.5%)	2564 (46.1%)	0%	0.153
**Age (years), mean (CI95)**		53.3 (52.9–53.7)	54.7 (53.9–55.4)	52.7 (52.2–53.2)	53.3 (53.0–53.6)	0%	**< 0.001**
**Weight (kg), mean (CI95)**		84.5 (83.5–85.4)	83.3 (81.8–84.9)	84.6 (83.6–85.7)	84.4 (83.7–85.0)	48.3%	0.372
**Height (m), mean (CI95)**		1.72 (1.72–1.73)	1.71 (1.70–1.72)	1.72 (1.71–1.73)	1.72 (1.72–1.72)	48.3%	0.137
**BMI (kg/m**^**2**^**), mean (CI95)**		28.4 (28.1–28.6)	28.4 (27.9–28.9)	28.5 (28.1–28.8)	28.4 (28.2–28.6)	48.4%	0.919
**Previous cervical spine surgery, count (%)**		332 (10.1%)	95 (13.3%)	167 (11.5%)	615 (11.0%)	1.9%	**0.029**
**Neck pain (VAS), mean (CI95)**		57.9 (56.5–59.3)	52.1 (49.4–54.8)	52.6 (50.9–54.4)	55.1 (54.1–56.1)	49.7%	**< 0.001**
**Upper limb pain (VAS), mean (CI95)**		59.9 (58.5–61.3)	54.4 (51.7–57.1)	54.3 (52.5–56.1)	57.1 (56.0–58.1)	51.3%	**< 0.001**
**NDI Score, mean (CI95)**		43.8 (42.9–44.7)	41.5 (39.7–43.2)	43.2 (42.1–44.2)	43.2 (42.6–43.9)	50.4%	0.062
**Working status pre-op, count (%)**						48.0%	**0.029**
	**Working**	732 (52.8%)	226 (47.4%)	526 (51.1%)	1484 (51.3%)		
	**Unemployed**	79 (5.7%)	40 (8.4%)	76 (7.4%)	195 (6.7%)		
	**Retired**	205 (14.8%)	93 (19.5%)	179 (17.4%)	477 (16.5%)		
	**Unable to work**	371 (26.7%)	118 (24.7%)	249 (24.2%)	738 (25.5%)		
**Pain duration pre-op, count (%)**						49.0%	0.965
	**Less than 6 weeks**	60 (4.4%)	22 (4.8%)	47 (4.7%)	129 (4.6%)		
	**6**–**12 weeks**	115 (8.4%)	41 (9.0%)	93 (9.2%)	249 (8.8%)		
	**3**–**12 months**	532 (38.8%)	180 (39.4%)	376 (37.3%)	1088 (38.4%)		
	**Over 1 year**	664 (48.4%)	214 (46.8%)	491 (48.8%)	1369 (48.3%)		
**Regular use of painkillers pre-op, count (%)**		842 (61.0%)	265 (58.0%)	582 (57.5%)	1689 (59.3%)	48.8%	0.179
**Cardiac comorbidity pre-op, count (%)**		106 (8.1%)	35 (9.8%)	81 (8.9%)	222 (8.6%)	53.8%	0.586
**Parkinson's disease pre-op, count (%)**		10 (0.8%)	1 (0.3%)	4 (0.4%)	15 (0.6%)	53.8%	0.436
**Smoking pre-op, count (%)**		372 (26.8%)	116 (24.7%)	210 (20.6%)	698 (24.3%)	48.4%	**0.002**
**Use of other nicotine products, count (%)**		128 (9.8%)	36 (9.7%)	76 (7.9%)	240 (9.1%)	52.5%	0.267
**Perioperative complication, count (%)**		54 (1.6%)	13 (1.8%)	29 (2.0%)	96 (1.7%)	47.0%	0.668
**Early complication, count (%)**		124 (3.7%)	39 (5.4%)	67 (4.5%)	230 (4.1%)	N/A^1^	0.078
**Late complication, count (%)**		17 (0.5%)	3 (0.4%)	19 (1.7%)	45 (0.8%)	N/A^1^	**<0.001**

Abbreviations.

CI95, 95% confidence interval; BMI, body mass index; VAS, visual analog scale; NDI, neck disability index.

^1^Missing data percentages for early and late complications are not applicable (N/A) as entries are only added in the register in case of an observed complication.

**Table 2:** Kuopio University Hospital subcohort baseline characteristics.

Baseline characteristic		FinSpine 12-month PROM/PREM's self-assessment surveys				p-value
		Not completed	Completed	Total	Missing	
**Patients, count (%)**		29 (48.3%)	31 (51.7%)	60 (100%)		
**Gender, count (%)**	**Female**	11 (37.9%)	20 (64.5%)	31 (51.7%)	0%	**0.039**
**Age (years), mean (CI95)**		55.2 (51.5–58.8)	56.7 (53.1–60.2)	56.0 (53.5–58.4)	0%	0.548
**Weight (kg), mean (CI95)**		81.5 (73.0–90.1)	81.5 (77.2–85.7)	81.5 (77.3–85.7)	43.3%	0.987
**Height (m), mean (CI95)**		1.74 (1.69–1.79)	1.70 (1.67–1.74)	1.72 (1.69–1.75)	43.3%	0.126
**BMI (kg/m**^**2**^**), mean (CI95)**		26.8 (24.3–29.2)	28.3 (26.5–30.1)	27.6 (26.2–29.0)	43.3%	0.289
**Previous cervical spine surgery, count (%)**		5 (17.2%)	6 (19.4%)	11 (18.3%)	0%	0.833
**Neck pain (VAS), mean (CI95)**		53.6 (38.6–68.5)	48.6 (30.4–66.8)	51.1 (40.1–62.1)	53.3%	0.650
**Upper limb pain (VAS), mean (CI95)**		53.6 (37.9–69.3)	57.1 (42.8–71.5)	55.5 (45.6–65.4)	53.3%	0.723
**NDI Score, mean (CI95)**		35.1 (25.4–44.7)	43.4 (33.1–53.7)	39.7 (32.8–46.6)	43.3%	0.229
**Working status pre-op, count (%)**					43.3%	0.280
	**Working**	9 (60.0%)	7 (36.8%)	16 (47.1%)		
	**Unemployed**	2 (13.3%)	1 (5.3%)	3 (8.8%)		
	**Retired**	1 (6.7%)	5 (26.3%)	6 (17.6%)		
	**Unable to work**	3 (20.0%)	6 (31.6%)	9 (26.5%)		
**Pain duration pre-op, count (%)**					50.0%	0.245
	**Less than 6 weeks**	2 (15.4%)	0 (0.0%)	2 (6.7%)		
	**6**–**12 weeks**	0 (0.0%)	2 (11.8%)	2 (6.7%)		
	**3**–**12 months**	5 (38.5%)	7 (41.2%)	12 (40.0%)		
	**Over 1 year**	6 (46.2%)	8 (47.1%)	14 (46.7%)		
**Regular use of painkillers pre-op, count (%)**		4 (26.7%)	12 (66.7%)	16 (48.5%)	45.0%	**0.022**
**Cardiac comorbidity pre-op, count (%)**		3 (18.8%)	1 (5.3%)	4 (11.4%)	41.7%	0.212
**Parkinson's disease pre-op, count (%)**		0 (0.0%)	0 (0.0%)	0 (0.0%)	41.7%	
**Smoking pre-op, count (%)**		6 (37.5%)	4 (21.1%)	10 (28.6%)	41.7%	0.283
**Use of other nicotine products, count (%)**		1 (6.3%)	0 (0.0%)	1 (2.9%)	41.7%	0.269
**Peroperative complication, count (%)**		0 (0%)	0 (0%)	0 (0%)	15.0%	
**Early complication, count (%)**		3 (13.6%)	4 (15.4%)	7 (14.6%)	N/A^1^	0.864
**Late complication, count (%)**		0 (0%)	0 (0%)	0 (0%)	N/A^1^	

Abbreviations.

CI95, 95% confidence interval; BMI, body mass index; VAS, visual analog scale; NDI, neck disability index.

^1^Missing data percentages for early and late complications are not applicable (N/A) as entries are only added in the register in case of an observed complication.

In the ACDF population of FinSpine, pre-operative smoking was associated with a significantly lower response rate (26.8% vs 20.6%, p = 0.002) while patients with a history of previous cervical spine surgery (11.5% vs 10.1%, p = 0.029) and patients who suffered a late post-operative complication (1.7% vs 0.5%, p < 0.001), were more likely to respond. Patients who responded more frequently had lower pre-operative VAS for neck (52.6 [50.9–54.4] vs 57.9 [56.5–59.3], p < 0.001) and upper limb (54.3 [52.5–56.1] vs 59.9 [58.5–61.3], p < 0.001) pain. Respondents were more frequently retired (17.4% vs 14.8%, p = 0.029) or unemployed (7.4% vs 5.7%, p = 0.029). A slight difference in age was observed, mean age of non-respondents was 53.3 [52.9–53.7], partial respondents 54.7 [53.9–55.4] and respondents 52.7 [52.2–53.2], p < 0.001.

### 3.2 Post-operative opioid purchases (SII)

Out of the 5563 FinSpine ACDF patients, 1362 (24.5%) purchased prescribed drugs containing opioids during 12-month post-operative period. Of these patients, 1173 had undergone primary ACDF and 189 non-primary ACDF surgery.

**Fig. 2:**
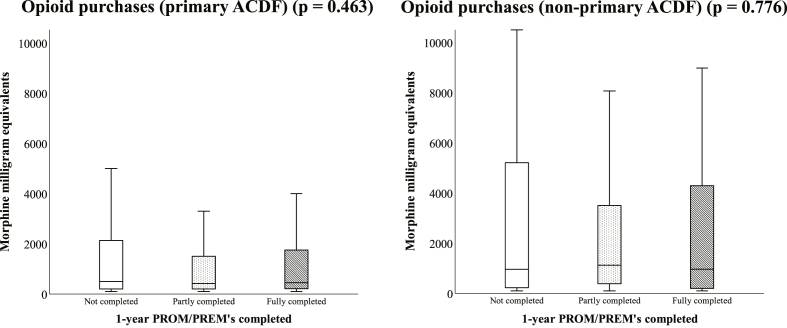
Comparison of opioid purchases in the FinSpine ACDF population of 5563 patients during the 1st post-operative year after primary ACDF surgeries and for patients that had previous cervical spine surgery. Patients were compared depending on their status of completing the 12-month post-operative self-assessment surveys used in FinSpine. Horizontal lines in boxplots represent the median purchased amount. P-values were obtained using the Kruskal-Wallis test. Outlier data points were excluded for a clearer range representation in the box plot.

There were no statistically significant differences in either patient group between respondents or non-respondents in median sum of opioid drug purchases made during 12-month post-operative period among patients who made at least a single purchase. In “Primary ACDF”-group, median cumulative purchased amount (in MME's) for non-respondents was 500 and interquartile range (IQR) 1951.3, for partial respondents 420, IQR 1306.0, and respondents 450, IQR 1566.0, p = 0.463. In “Non-primary ACDF”-group, median for non-respondents was 960, IQR 5017.5, partial respondents 1125, IQR 3636.0, and respondents 964 IQR 4450.0, p = 0.776. Box plots of sums of opioids purchased are shown in Fig. 2.

### 3.3 Outcome measures (subcohort questionnaire)

**Fig. 3:**
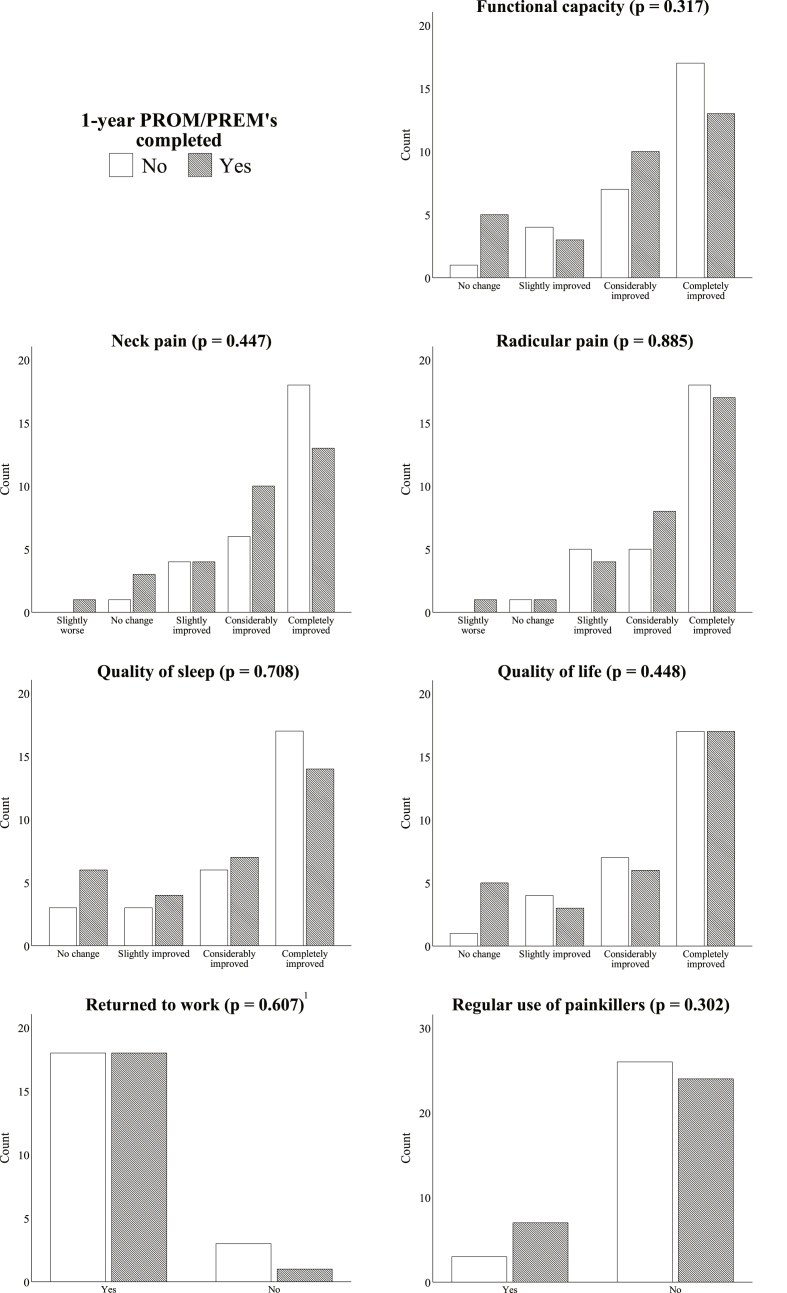
Outcome measures of 60 consecutive patients who underwent ACDF surgery at Kuopio University Hospital (FinSpine subcohort) between August 1st, 2019, and December 31st, 2019, according to 12-month follow-up questionnaire. Patients were compared depending on their status of completing the 12-month post-operative self-assessment surveys used in FinSpine. P-values were obtained using Fisher's exact test.^1^Outcomes of patients who were working prior to the ACDF surgery (n = 40).

Twelve-month post-operative outcome measures for the subcohort are shown in Fig. 3. No statistically significant differences were observed in any of the outcome measures between respondents and non-respondents.

Radicular upper-extremity pain resolved completely in 62.1% of non-respondents and 54.8% of respondents, while considerable improvement in radicular symptoms was observed in 17.2% vs 25.8%, respectively (p = 0.885). 89.7% of non-respondents and 77.4% of respondents did not need painkillers regularly at 12 months after the surgery (p = 0.302). Out of 40 patients that were working prior to ACDF surgery, 85.7% of non-respondents and 94.7% of respondents returned to work (p = 0.607).

## 4 Discussion

Our study population was derived from 48600 patients identified in FinSpine, of whom 5563 underwent ACDF surgery. Before interpreting outcome measure results and differences between respondents and non-respondents at 12 months, we studied their purchases of prescribed opioids during first post-operative year in order to validate outcome results using a large sample size, objective variable data that was not previously linked to FinSpine and found no difference between patients who had responded to FinSpine post-operative self-assessment surveys at 12 months and those who had not. We further analyzed a subpopulation of a single center, consecutive patients who underwent ACDF surgery and found no differences in outcome measures at 12 months between the same groups. Whether individuals responded to follow-up or not, their return-to-work rates were comparable, and there was no notable difference in the regular use of pain medication. Similar improvements in upper-extremity radicular pain and functional capacity were observed across the entire subcohort. The findings from the subcohort indicate that satisfied patients do not disproportionately represent the data in FinSpine, as while non-significant, all outcome measure results in subcohort lean towards respondents reporting marginally less favorable results.

While there was no significant difference in clinical outcomes at 12 months, some differences in baseline variables were observed. In the FinSpine population, non-smoking patients, as well as those who initially reported less severe upper limb or neck pain, were more likely to respond to the surveys. It was also observed that respondents more frequently suffered from post-operative complications or had a history of cervical spine surgery. Although there was a slight variation in age among the groups, the slight difference in age could be interpreted as negligible, as the mean age for all groups fell within a 2-year range, with partial respondents being the oldest, and patients who completed all questionnaires being the youngest.

Smoking and male gender have been previously associated with non-responding in spine register studies (Patel et al., 2022; Parai et al., 2020; Ingebrigtsen et al., 2023). The higher response rates among patients with prior cervical spine surgery could be due to increased awareness of the surveys. Additionally, patients who suffered complications after surgery might have been more inclined to respond due to the ongoing effect of the surgery on their daily lives, particularly if the resulting outcome was unfavorable.

To our knowledge, only one study has been previously published addressing non-respondent bias in cervical spine surgery using data from national, clinical-quality register. In this Norwegian spine register (NORSpine) study, Ingebrigtsen et al. described a similar study setting for a smaller subpopulation cohort, comparing respondents and non-respondents. While their cohort was larger, they did not manage to interview 34.3% of non-respondents (Ingebrigtsen et al., 2023). Findings in the cervical spine NORSpine study and three other Nordic national clinical quality register studies focusing on lumbar spine surgery suggest similar results with no significant differences in outcome measures between respondents and non-respondents (Elkan et al., 2018; Højmark et al., 2016; Solberg et al., 2011).

While a total response rate to post-operative 12-month PROM and PREM questionnaires for the FinSpine ACDF population was 39.6%, yearly average response rates have been steadily climbing year-by-year, being currently at 54–58%. Different approaches have been reported to improve completion compliance. In 2019, in the United Kingdom, a tariff was introduced to encourage data completion in the British Spine Registry, but the tariff didn't extend to cover PROM/PREM questionnaires (Habeebullah et al., 2021).

Response rates within FinSpine vary significantly among participating centers. A few centers began participating in FinSpine without immediately implementing the PROM/PREM section, resulting in data that contained solely non-respondents from that period. Currently, to address lower response rates, more centers are appointing dedicated research coordinators to oversee the PROM/PREM response process. These efforts have led to an impressive increase in response rates, with the top-performing centers achieving 90% pre-operative and 80% post-operative response rates (Marjamaa et al., 2023).

The main strength and novelty of the current study is the use of the prospective national prescription drug register. Opioid purchases can be used as an objective surrogate outcome measure, that has no loss to follow-up and combined with the national dataset of FinSpine, covers all Finnish hospitals performing ACDF surgery. However, we must acknowledge the limitation that while the prescription drug data is complete, it does not allow us to differentiate the specific reasons for each drug purchase. Another key strength of this study is the integrity of data from the single center subcohort. Despite its relatively small size, all non-respondents were reached and interviewed, enabling a genuine comparison between respondents and non-respondents.

## 5 Conclusion

Our results suggest that non-respondents do not skew the assessment of outcome measures following ACDF surgery at 12 months. Although it is crucial for all registers to aim for the lowest possible data attrition, the data from national clinical-quality registers like FinSpine appear to be valid and reliable. It can be argued that the outcome measure results derived from it represent the entire register population. Therefore, FinSpine and other quality register data can and should be used for further clinical research to improve patient care and to help allocate limited resources, especially in publicly funded healthcare systems.

## Statements and declarations

Nikolai Klimko received a working grant from Maire Taponen – foundation.

No other funding was received to assist with the preparation of this manuscript.

The authors have no competing interests to declare that are relevant to the content of this article.

All authors have agreed to the conditions noted in the journal's copyright agreement.

The datasets generated and analyzed in the current study can be accessed upon approval of a permit application submitted to the Finnish social and health data permit authority, Findata, in accordance with the Finnish law on the secondary use of health and social data.

This register study has been permitted by the Finnish Institute for Health and Welfare with the approval from the Ministry of Social Affairs and Health of Finland.

## Declaration of competing interest

The authors declare the following financial interests/personal relationships which may be considered as potential competing interests: Nikolai Klimko reports financial support was provided by Maire Taponen Foundation. If there are other authors, they declare that they have no known competing financial interests or personal relationships that could have appeared to influence the work reported in this paper.
