# Supplementary figures and images for: Outcome measures after anterior cervical decompression and fusion surgery –non-respondents do not bias the results: A Finnish spine register (FinSpine) study
Source: Brain Spine. 2024 Dec 31;5:104179. doi: 10.1016/j.bas.2024.104179 (PMC12092338; doi:10.1016/j.bas.2024.104179)

## Slide 1
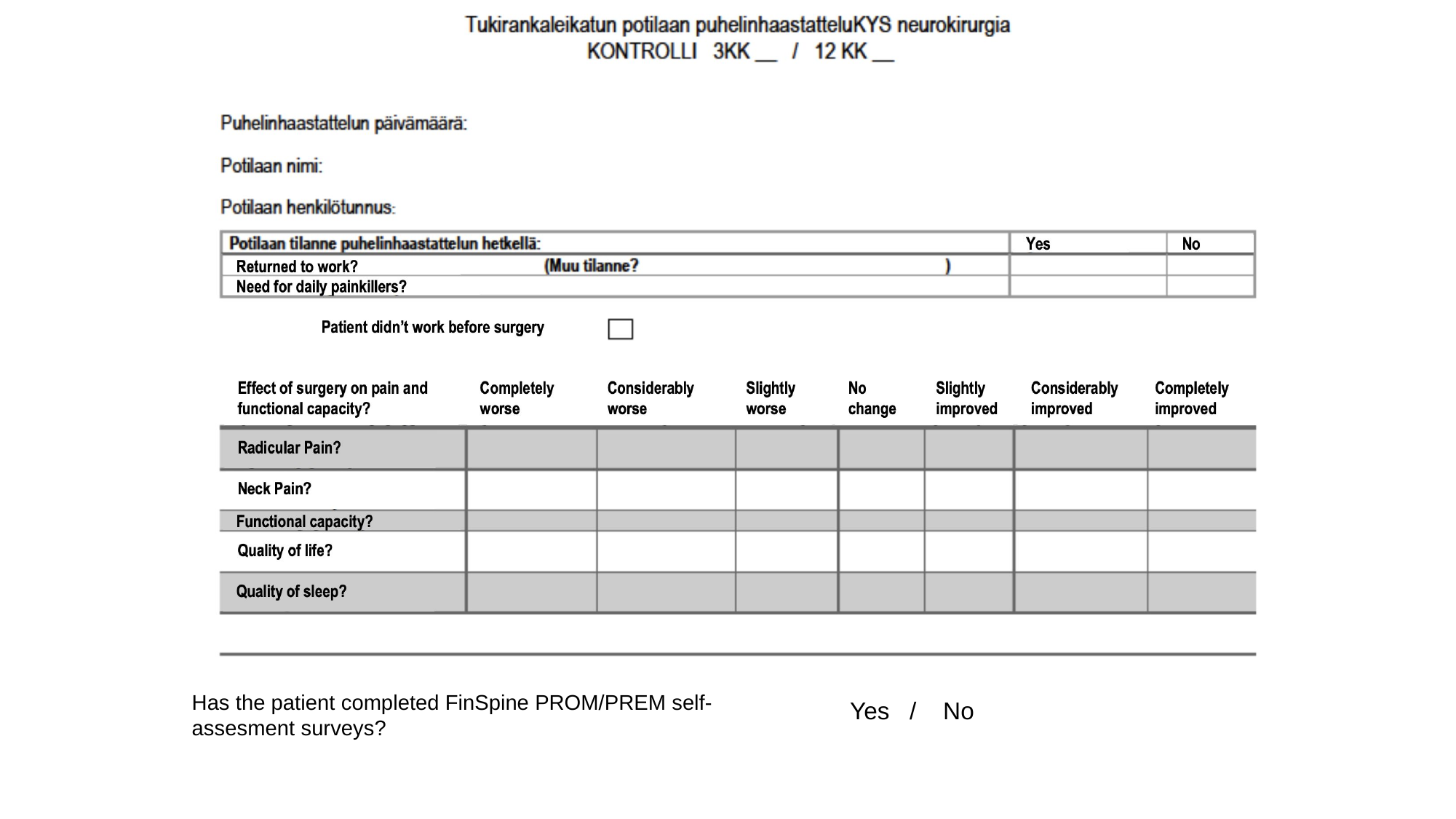

Has the patient completed FinSpine PROM/PREM self-assesment surveys?
Yes / No

Supplement: Multimedia component 1 [file mmc1.pptx]
